# Supplementary material for: MetaRibo-Seq measures translation in microbiomes
Source: Nat Commun. 2020 Jun 29;11:3268. doi: 10.1038/s41467-020-17081-z (PMC7324362; doi:10.1038/s41467-020-17081-z)
Supplement: Supplementary file 10 — Supplementary Data 7 [file 41467_2020_17081_MOESM10_ESM.zip › File2/Confidence_High_Taxonomy/89566_out.krona.html]

Javascript must be enabled to view this page.

members
magnitude
magnitudeUnassigned
count
unassigned
taxon
rank

89566\_out

3

superkingdom
3
2

1239
3
phylum

91061
3
class

order
3
186826

1300
3
family

genus
1301
3

species

SRS018573\_contig\_number\_1659SRS045197\_contig\_number\_contig-100\_61.225196SRS104521\_contig\_number\_25661
1309
3
